# Natural Resources Exploitation in Sulfate-Resisting Portland Cement Manufacturing: Towards Quality Improvement and Reduction of Environmental Impact

**DOI:** 10.3389/fchem.2022.806433

**Published:** 2022-08-17

**Authors:** Islem Labidi, Adel Megriche

**Affiliations:** Laboratoire de Chimie Minérale Appliquée LRCMA (LR19ES02), Faculté des Sciences de Tunis, Université de Tunis El Manar, Campus Universitaire Farhat Hached El Manar, Tunis, Tunisia

**Keywords:** SR Portland cement-C_3_A, manufacturing process-stability, CO_2_ emission, burning modules of raw meals, calculation tool

## Abstract

Sulfate-resisting **(**SR) Portland cement is commonly used in building works to improve concrete’s durability against external sulfate attack. This attack is considered a very serious chemical aggression that causes damage and cracking of concrete structures. These special cements have a very particular mineralogical composition, C_3_A ≤ 3% and (2C_3_A + C_4_AF) ≤ 20%, which makes the cementitious matrix resistant to sulfate attack. This kind of product is very difficult to manufacture since low alumina (C_3_A) necessitates the use of a high kiln temperature in order to keep a sufficient liquid phase necessary to maintain the stability of the cement manufacturing process. In this context, this study aims to optimize SR Portland cement raw meals using natural materials collected from different regions in Tunisia, mainly ordinary limestone, siliceous limestone, black marl, grey marl, iron ore, and natural fluorapatite. The collected specimens were characterized by an X-ray fluorescence spectrometer in order to determine its elemental chemical composition. The optimization of the SR Portland cement raw meal combinations was done by means of a calculation tool based on the chemical composition of each used raw material and the variation of burning modules (LSF, SIM, and ALM). It has been found that natural fluorapatite integration (0%–15%) in raw mix preparation leads to the raw meals required for the SR Portland cement standard (C_3_A ≤ 3% et 2 C_3_A + C_4_AF ≤ 20%). Moreover, it was shown that the estimated SR raw meals ensure the cement manufacturing process stability (acceptable burning modules “LSF = 100; SIM = 3; ALM = 0.91 and sufficient liquid phase) and decrease the CO_2_ emissions in cement production.

## 1 Introduction

Industrial and technological progress provide the basis for unrestrained growth in all fields, and in particular the building sector. Cement, undoubtedly, is considered an essential building block and it is a sign of the economic and social development of countries.

Cement is formed basically from an artificial stone called Portland clinker. The latter is obtained from the burning at a very high temperature, above 1,450°C, of a mixture of natural materials, mainly limestone and clay. The clinker is characterized by a very special composition and four main mineralogical phases: alite (C_3_S), belite (C_2_S), aluminate (C_3_A), and ferrite (C_4_AF) (Telschow et al., 2012; Ghosh et al., 2022). The cement generally used in the design of construction work is characterized by mechanical strength, although the interaction between concrete and the environment is often neglected. These environments can induce concrete deterioration either by physical attacks through surface erosion, freezing/thawing, and heat, or by chemical aggressions such as acids, sulfates, alkali-aggregate reactions, and steel corrosion (Association, 2002). All these phenomena have a negative impact on concrete durability (Al-Amoudi, 2002). Sulfate ions’ penetration in cement matrix presents a fairly dangerous pathology for concrete due to its expansive consequences. This kind of attack was observed for the first time in 1887 by Candolt during their investigation into mortars in the fortifications of the city of Paris (Ragoug, 2016). Indeed, these mortars were in contact with aggressive waters rich in sulfate ions. The mitigation of external sulfate attack is considered a necessity to maintain the concrete’s durability. So, many methods were adopted to minimize external sulfate attack (Kanaan et al., 2022), which are based on the use of mineral additives as partial cement replacement, such as silica fume, blast furnace slag, fly ash, metakaolin, pozzolana natural, and limestone. The European standard “Cement—Part 1: composition, specifications, and conformity criteria for common cements” (CEN, Cement, 2011) introduces a family of sulfate-resisting common cements (SR-cements). Certain types of these cements, such as CEMIII/B-SR, CEMIII/C-SR, CEMIV/A-SR, and CEMIV/B-SR, pose difficulties during their production in certain situations, such as in Tunisian cement plants, since they require blast furnace slag, natural pozzolana, and siliceous fly ash, and the availability of these materials is very delicate. So, in this case, the only way to combat the external sulfate attack is the use of sulfate-resisting (SR) Portland cement. SR Portland cement is a hydraulic binder used in sulfate-rich construction environments (Neville, 1995) to protect the concrete from external sulfate attack. These cements have a very special mineralogical composition: C_3_A ≤ 3% and 2C_3_A + C_4_AF ≤ 20% (NT, 47.26, 1999).

SR Portland cement production is very complicated since low aluminate content necessitates the use of high kiln temperature (Consumption of enormous energy) in order to keep a suitable liquid phase (LP) required to maintain clinkering stage stability (Labidi et al., 2019a; Dorn et al., 2022).

In fact, the cement industry used two tricks to limit the alumina content in SR Portland cement during its manufacturing:

• The first method involves using limestone as a partial clinker replacement during the cement milling process to artificially lower the C_3_A content. But this behaviour affects the durability of SR Portland cement (Labidi et al., 2019a), since limestone in contact with sulfate ions induces the thaumasite formation. This leads to concrete deterioration (Schmidt, 2007; Schmidt et al., 2009).
• The second method deals with the increase of iron ore raw material at the moment of raw mix preparation, which is considered an apparent industrial solution. But in reality, this practice can cause a serious disequilibrium of the cement process and also affect the cement mineralogy (Chatterjee, 2011; Sorrentino, 2011).

It seems that studies dealing exclusively with the optimization of SR Portland cement raw meal formulas from Tunisian natural raw materials, especially without iron ore increasing, are not being conducted. The aim of the present research is to optimize different raw meals for sulfate-resisting Portland cement from natural resources (ordinary limestone, siliceous limestone, black marl, grey marl, iron ore, and natural fluorapatite) collected from different locations in Tunisia. These optimized combinations are expected to meet SR Portland cement standards, guarantee the stability of the cement production process and, above all, reduce the environmental impact, since cement manufacturing is the third most CO_2_-emitting industrial sector in the world (652 kg–894 kg CO_2_/t_cement_) (Altun, 1999a; Kajaste and Hurme, 2016; Mishra et al., 2022). This work is done essentially by means of a raw meal calculation program based on the chemical composition of the raw materials.

The chemical composition analysis of each raw material was determined by X-ray fluorescence (XRF).

This paper is subdivided into three major sections: the first one presents an overview of the SR Portland cement manufacturing process, its mineralogical phases, its standards, and its chemical specifications. Following this, it was necessary to present and discuss the mechanisms of external sulfate attack. In other words, this will explain the usefulness of SR Portland cement in the fight against external sulfate attacks. The second presents the materials and methods used to carry out this research study, followed by the third section involving the results and discussions of different studied cases of SR Portland cement raw meal combinations optimization.

## 2 Bibliographic Review

### 2.1 Sulfate-Resisting Portland Cement

SR Portland cement is a hydraulic binder used for massive structures exposed to aggressive environment construction works (Essawy and Abd El.Aleem, 2014). Its manufacturing is similar to ordinary Portland cement. It is characterized by a low content of aluminate phase (0%–5%) compared to ordinary Portland cement of about 12%.To produce this kind of binder, most cement industries increased the iron oxide (iron ore) content in their raw meals for purposes of obtaining a clinker with a small amount of C_3_A (Essawy and Abd El.Aleem, 2014). This behaviour induces the decrease of the clinker liquid phase noted L.P (or clinker melt) viscosity during raw mill burning since the liquid phase viscosity increases linearly with the alumina ratio (ALM = Al_2_O_3_/Fe_2_O_3_).

The amount of L.P at 1,450°C Eq. (A.1) is computed according to the relation between the Al_2_O_3_, Fe_2_O_3_, MgO, K_2_O, Na_2_O, and SO_3_ contents (Mosci, 2000).

$$\%L.P.\;at\;1450\;\text{°C}=3\times \text{A}+2\text{.}25\times F+M+K+N+\overset{}{S}\left(MgO\leq 2\right)\;$$ (A.1)

**FIGURE 1:**
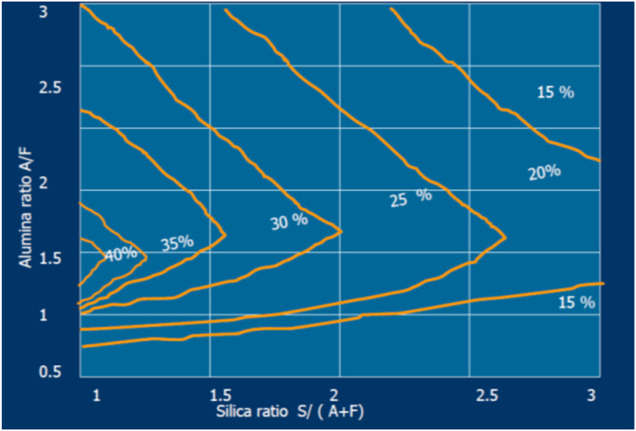
Variation in % liquid phase at 1,338°C with change in silica ratio (SIM = S/[A + F]) and alumina ratio (AR) at LSF = 100, LSF = C/[2.8S + 1.1 A + 0.7F] (Kumar, 2013).

**FIGURE 2:**
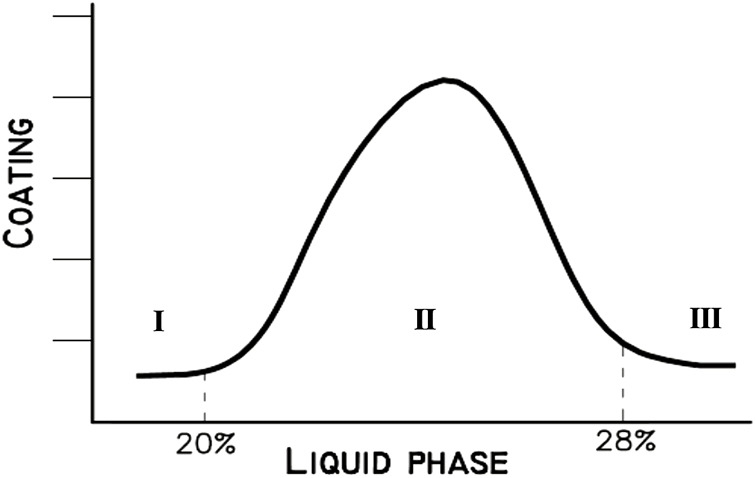
Liquid phase % versus coating formation (I: L.P is not a sufficiently pasty form, II: Pasty form of L.P, and III: L.P is very fluid-liquid) (Sengupta, 2020).

Moreover, the quantity of liquid phase depends strongly on the burning modulus (ALM and SIM) variation as presented in Figure 1 and, at the same time, it controls the coating formation (see Figure 2).

The latter protects the refractory bricks of the cement kiln and its continuance is considered a necessity (Belgacem et al., 2016). Indeed, as the viscosity of L.P is increased, the coating will be heavier and the risk of brick removal will be increased. Figure 2 gives three different intervals:

• %L.P < 20: The formed liquid phase is not sufficiently pasty and is regarded as very low. In this case, alite (the most important clinker mineral and the controller of the mechanical strength development of the concrete at an early stage) formation is extremely slow and difficult since the liquid phase creates the reaction medium for the conversion of C_2_S to C_3_S. The formed coating is thin, weak, not stable, and has a rough surface.
• 20% < %L.P < 28%: The liquid phase has a pasty form and it is suitable for mineralogical phases’ formation. The coating is strong and stable, and the refractory bricks are almost protected.
• %L.P > 28%: The liquid phase is very fluid and there is a high risk of ring formation in the kiln, which causes the blockage of the burning lining.

On the one hand, the liquid phase has a serious role in clinker nodulization and clinker mineralogical phases’ formation. In the absence of the liquid phase, the reaction between C_2_S and free lime (CaO) to generate an alite phase (C_3_S) would be almost impossible during clinkerization. On the other hand, for a given burning temperature, high C_3_A clinkers tend to nodulize better than low C_3_A clinkers.

So, it is imperative to optimize a good raw meal (especially one that is not doped with iron ore) to obtain SR Portland cement with a low amount of aluminate and to prevent poor operation of the cement kiln.

Sulfate-resisting Portland cement is a special purpose hydraulic binder used where sulfates are present in high concentrations that would damage concrete formed by ordinary Portland cement. External sulfate attack strength is achieved by setting the mineralogical composition to limit the amount of aluminate compound (C_3_A) in the sulfate-resisting Portland cement. Indeed, the degradation rate of concrete exposed to aggressive sulfate environments depends essentially on C_3_A content (Adedoud, 2019). The lower the quantity of aluminate, the more sulfate resistance is increased (Nawy, 2008). This parameter is considered the most important characteristic of sulfate-resisting Portland cement.

The quality and performance of all sulfate-resisting Portland cement produced by any cement industry in the world must conform to the characteristics and specifications of SR Portland cement as indicated in the standard. Table 1 summarizes the main sulfate-resisting Portland cement standards.

**TABLE 1:** Sulfate-resisting Portland cement standards.

	Standards	Designations of the sulfate resisting Portland cement		Requirements		
				Mineralogical	Chemical	Medium specifications
European standard (EN)	EN197-1:2011: cement-Part 1: Composition, specifications, and conformity criteria for common cements (CEN, Cement, 2011)	Sulfate resisting Portland cement	CEMI-SR0	C_3_A = 0	—	—
			CEMI-SR3	C_3_A ≤ 3%		
			CEMI-SR5	C_3_A ≤ 5%		
American society for testing and method standard (ASTM)	ASTM C 150/150M-19a: Standard specification for Portland cement (ASTM, 2017)	Sulfate-resisting Portland cement: Type V		C_3_A ≤ 5%	SO_3_ ≤ 2.3%	—
				2 × C_3_A + C_4_AF ≤ 25%	MgO ≤ 6%	
British standard (BS)	BS 4027:1996-Specification for sulfate resisting Portland cement (Hooton and Thomas, 2002)	Sulfate resisting Portland cement: SRPC		C_3_A ≤ 3.5%	SO_3_ ≤ 2.5%	—
				2 × C_3_A + C_4_AF ≤ 25%	MgO ≤ 4%	
					Cl^−^ ≤ 0.02%	
Tunisian standard (NT)	NT 47.26 (1998) (NT, 47.26, 1999)	High sulfate resisting Portland cement:HRS1		C_3_A ≤ 3%	SO_3_ ≤ 3.5%	−[SO_4_^2−^] > 600 mg/L in solutions
				2 × C_3_A + C_4_AF ≤ 20%	MgO ≤ 4%	−%SO_3_ > 2.4% in dry soils
		High sulfate resisting Portland cement: HRS2		3% ≤ C_3_A ≤ 5%	SO_3_ ≤ 2.5%	−1,500 < [SO_4_^2−^] < 6,000 mg/L in solutions
				2 × C_3_A + C_4_AF ≤ 20%	MgO ≤ 4%	−1.2% < %SO_3_ < 2.4% in dry soils

Moreover, the Tunisian standards [NT 47.26 (1998) and NT 47.25 (1998)] (NT, 47.26, 1999) introduced the classification of severity of sulfate environments:

- Sulfate-rich environments:
  • Solutions: the concentration of sulfate ions is greater than or equal to 1,500 mg/L;
  • Soils: the sulfate ion amounts are greater than or equal to 1.2%.
- Sulfate environments of moderate severity:
  • Solutions: the concentration of sulfate ions is situated between 600 mg/L and 1,500 mg/L,
  • Soils: the sulfate ion amounts are situated between 0.6% and 1.2%.

The quantification of aluminate (C_3_A) and ferrite (C_4_AF) phases as indicated in European standard EN 197-1 is based on the Bogue calculation method (Labidi et al., 2019b).

Bogue calculation method remains a primary estimation of the major cement phases. This tool is applied only in the perfect conditions of clinker burning, in the absence of minor’s elements: all Fe_2_O_3_ quantity reacted with a part of Al_2_O_3_ and lime to form the C_4_AF and the residual alumina is combined with CaO to make aluminate phase. However, this assumption does not take account of Fe_2_O_3_ and Al_2_O_3_ insertions in the alite and belite phases during the real clinkering process (Labidi et al., 2019b). Thus, the aluminate and ferrite amounts by using Bogue method probably contained an error and should be revised or another more accurate means should be used to quantify C_3_A and C_4_AF such as X-rays diffraction coupled with Rietveld refinement (Mohamed et al., 2017).

So, the ASTM C150/150M-19a (ASTM, 2017), BS 4027:1996, and NT 47.26 (1998) imposed a second condition “2 × C_2_A + C_4_AF” to limit the quantity of iron oxide in the raw mill. It seems that this requirement is stricter in Tunisian standards than the other standards.

### 2.2 Why the Use of SR Sulfate Portland Cement is Necessary?

#### 2.2.1 External Sulfate Attack

**FIGURE 3:**
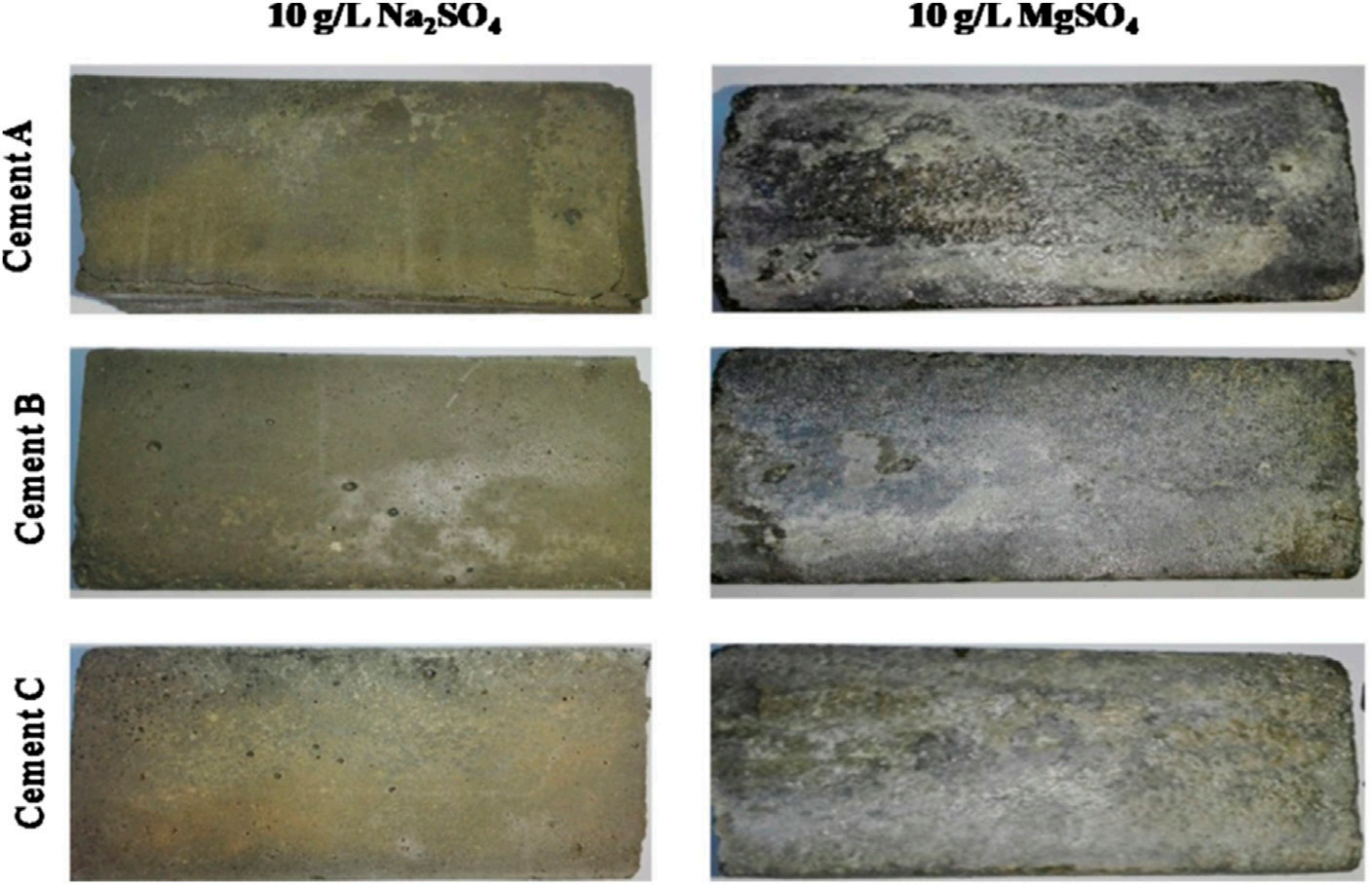
Photograph of mortars of different samples of Tunisian sulfate-resisting Portland cement conserved for 2 years in 10 g/L Na_2_SO_4_ and 10 g/L MgSO_4_ (Labidi et al., 2019a).

The durability of concrete structures can be significantly improved if the effects of the surrounding environment are taken into account ahead of the formulation of the material and the dimensioning of the structure. Despite the various studies and expertise, some degradation mechanisms remain unclear and controversial. This is the case, for example, with external sulfate attack (ESA) or external sulfate reaction. Sulfate ions can originate from groundwater (Lothenbach et al., 2010), soils rich in gypsum or pyrite (Schmidt et al., 2009), sulfates from industrial products, fertilizers or organic substances, river water, or sea water (Aziez, 2017). It is also important to note that dry salts do not react with concrete. The presence of sulfate in solution is essential for the transfer of SO_4_^2−^ ions into the concrete matrix (Neville, 2004). The external sulfate attack is associated with the precipitation of secondary sulfate products, expansions, and the deterioration of physicochemical properties of concrete, which induces the loss of strength and cohesion, and even the scaling, cracking, and disintegration of the cement matrix (see Figure 3).

#### 2.2.2 External Sulfate Attack Mechanisms

The mechanisms of external sulfate attack have been largely studied and reviewed (Al-Amoudi, 2002; Whittaker and Black, 2015) since 1983 (Mehta, 1983). So, this section explains briefly the different steps of the ESA process.

The external sulfate attack depends on the type of cation associated with sulfate ions. In fact, sulfate ions can be found in the environment as Na_2_SO_4_, MgSO_4_, K_2_SO_4_, CaSO_4,_, and (NH_4_)_2_SO_4_ (Xiong et al., 2014).

In this part, the attack mechanisms in sodium sulfate and magnesium sulfate environments are described in Table 2 since they are considered the most common sulfate attack studied (Collepardi, 2001; Neville, 2004; Page and Page, 2007; Rheinheimer, 2008; Irassar, 2009; Whittaker and Black, 2015; Sengupta, 2020) and reviewed around the world.

**TABLE 2:** The sodium sulfate and magnesium sulfate attacks mechanisms.

External sulfate attack types	Attack mechanisms	Description and consequences
Sodium sulfate attack (Al-Amoudi, 2002)	Ca(OH)_2_ + NaSO_4_ + 2H_2_O → CaSO_4_.2H_2_O + 2 NaOH (R°1) Ca_3_Al_2_O_6_ + 3CaSO_4_.2H_2_O + 26H_2_O → Ca_6_Al_2_(SO_4_)_3_(OH)_12_·26H_2_O (R°2)	• The secondary gypsum (CaSO_4_.2H_2_O) caused an increase in volume and a loss of rigidity and force to the concrete, thereby increasing the degradation (Rondeux et al., 2014)
		• The secondary ettringite (Ca_6_Al_2_(SO_4_)_3_(OH)_12_·26H_2_O) has a hazardous effect on the cementitious materials since it grows in the concrete in the form of needles. The latter induces the expansion, deterioration, and even breaking of the concrete structure (Rondeux et al., 2014)
Magnesium sulfate attack (Santhanam et al., 2003)	Ca(OH)_2_ + MgSO_4_ + 2H_2_O → CaSO_4_.2H_2_O + Mg(OH)_2_ (R°3) Ca_3_Al_2_O_6_ + 3CaSO_4_.2H_2_O + 26H_2_O→Ca_6_Al_2_(SO_4_)_3_(OH)_12_·26H_2_O (R°5)	• The magnesium sulfate attack induced the disintegration or decalcification of the cement matrix by the conversion of C-S-H to M-S-H (Labidi et al., 2019a).Therefore, the concrete will lose its binding character since the magnesium silicate hydrate (M-S-H) is a non-cementitious materials
		• This attack is more damaging than sodium sulfate aggression, since it is characterized by softening and deterioration of the superficial layers of the hardened cement paste (Rasheeduzzafar et al., 1994; Al-Amoudi, 2002)

The cement matrix is characterized by the presence of hydrated phases that results from the hydration of mineralogical phases of cement. Indeed, during cement hydration, calcium silicate hydrate named C-S-H, and portlandite or calcium hydroxide [Ca(OH)_2_] are formed from the alite and belite dissolution. Aluminate compound reacts with gypsum, which is added to the cement during clinker milling, to form the primary ettringite (C_3_A. 3C $\bar{S}$ 32H) and the monosulfoaluminate (C_3_A. C $\bar{S}$ 12H). For the ferrite phase (C_4_AF), it adopts the same mechanism as the aluminate phase hydration. Calcium silicate hydrate is the majority phase in the cement concrete, and it participates in the development of mechanical strength and the maintenance of the particles’ adhesion to the cement concrete.

Because the portlandite (CH) content in cement paste plays a significant role in the progression of the sulfate attack mechanism (De Souza et al., 2018; De Souza et al., 2020), it is preferable to use a sulfate-resistant Portland cement that produces a minimum amount of portlandite (a small amount of C_3_S) in the system and contains a lower quantity of aluminate phase (Labidi et al., 2019a).

**FIGURE 4:**
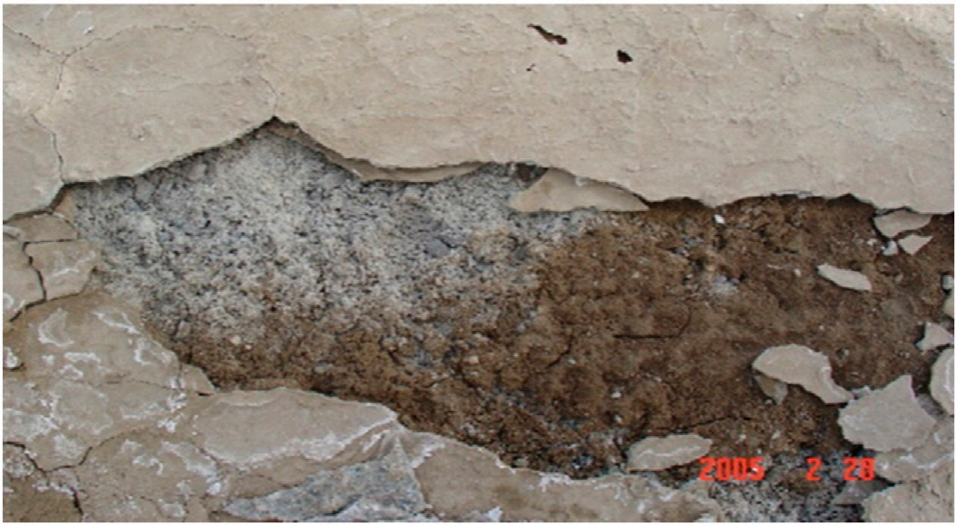
Thaumasite formation under external sulfate attack (groundwater [SO_4_^2−^] = 653 mg/L) in the Yongan Dan in Keshi of China (Mingyu et al., 2006).

Another consequence of the external sulfate attack is the thaumasite “ $C_{3}\bar{CS}SH_{15}$ ” formation, also called the thaumasite sulfate attack (TSA), considered a serious danger to concrete durability, especially in cold environments, since it causes concrete degradation. This kind of attack has attracted a great deal of attention in the world since 1995 in the United Kingdom (Crammond and Halliwell, 1995); this issue has been observed in many regions around the world, such as Yongan Dam in Keisha, China, in February 2005 (Mingyu et al., 2006) (see Figure 4). Thaumasite formation requires the presence of sulfate ions, a carbonate source, and a low temperature (<15°C) (Rahman and Bassuoni, 2014). This kind of attack has been observed in Tunisian SR Portland cements mortars immersed in aggressive sulfate solution for 2 years (Labidi et al., 2019a).

The thaumasite is the result of two possible routes:

- Thaumasite is formed from secondary ettringite [Ca_6_Al_2_ [(OH)_4_SO_4_]_3_.26H_2_O] by replacing aluminate ions Al^3+^ with silicate ions Si^4+^ and the interstitial substitution of [(SO_4_^2−^)_3_(H_2_O)_2_] by [(SO_4_^2−^)_3_(CO_3_^2−^)_2_] (Ramachandran et al., 2002):Ca_6_ [Si(OH)_6_]_2_(CO_3_)_2_(SO_4_)_2_.24H_2_O = CaCO_3_.CaSO_4_.CaSiO_3_.15H_2_O = $C_{3}\bar{CS}{\mathrm{SH}}_{15}$ .
- The second mechanism to obtain the thaumasite product, an interaction between sulfate ions, carbonates, and the C-S-H gel, as mentioned in the following reaction (R°5) (Torres et al., 2003):

$$C\bar{S}H_{2}+C\bar{C}+C-S-H+12H\to C_{3}\bar{CS}SH_{15}\left(\text{R°}5\right)$$

Another important external sulfate attack studied using the ammonium sulfate solution (Girardi and Maggio, 2011; Martins et al., 2021) presents a deterioration of concrete due to the secondary gypsum and secondary ettringite formation (Brown, 2002), similar to Na_2_SO_4_ and MgSO_4_ mechanism attacks. Moreover, the C-S-H degradation was detected in this chemical aggression type (Marchand et al., 2001; Martins et al., 2021), which may eventually convert into amorphous hydrated silica (R°6). This external attack caused cracks and decomposition of concrete mainly in the interfacial zone (Girardi and Maggio, 2011).

$$xCa.SiO_{2}aq+x{\left(NH_{4}\right)}_{2}SO_{4}+xH_{2}O\to SiO_{2}.aq+xCaSO_{4}.2H_{2}O+2xNH_{3}\left(\text{R°}6\right)$$

## 3 Materials and Methods

### 3.1 Materials

In this study, seven raw materials were selected for the optimization of the raw meal combinations:

• Ordinary limestone, grey, and black marls: these raw materials are used in the production of CEM I and CEMII cement types at the Bizerte cement plant “SCB.” This cement industry is located in BP 53- Sebra bay, Bizerta-Tunisia.
• Siliceous limestone, yellow marl, black limestone, and flint: These natural resources are located in abandoned deposits of the SCB quarry. These materials are not used in cement production within the Bizerte cement factory. The used siliceous limestone is characterized by its pink color, and the flint is a hard sedimentary rock rich in silica oxide.
• Iron ore: It is considered a corrective material essentially used in Portland cement production in the SCB plant, which is extracted from the El Harach Tamara ore in Nefza-Tunisia.
• Natural fluorapatite: This natural material is exploited from the phosphates deposits of the Mdhilla region of the Gafsa phosphate company (CPG) in the southeast of Tunisia. The CPG is a Tunisian phosphate mining company based in Gafsa.

#### 3.1.1 Sampling and Treatment

As mentioned previously, some of the raw materials used in the Sulfate Resisting Portland cement Raw Meals (SRRM) optimization are derived from the SCB plant’s own quarries (Bir Massiougha, Jbel Abiodh, and Jebel Baccar deposits). These quarries are located 3.5 km west of Bizerte city and extend over an overall area at about 176 ha.

Limestone is extracted from the rock walls of an open pit quarry. Rock blasting is assured by using explosives. The resulting materials are transported to the crushing unit by dumper trucks.

The marl is extracted by means of wheel loaders directly from the deposit without blasting. The excavated material is transported to the processing site by transport vehicles.

Thus, the sampling of limestone and marl samples was carried out in the quarries of the SCB factory under the guidance of a consulting geologist in order to take a minimum quantity of 2.5 kilos of each sample (limestone, marl, and flint).

The choice of these materials is based on their availability in significant quantities in the SCB quarries, according to the various operation surveys conducted by the Bizerte cement plant.

In this study, sample treatment is considered a key operation in order to guarantee accurate and reliable X-ray fluorescence analysis results.

In the first step, each raw material is crushed to a particle size of less than 5 mm by means of a jaw crusher in order to eliminate the size factor. The latter is a guiding vector for the variation of the crushing material fineness.

In the second step, each sample undergoes a drying operation (in the oven for 24 h at 100°C ± 5) to remove the water from moisture, which subsequently facilitates secondary grinding. This grinding was carried out with a grinder-shaker on hold of about 250 g of the powder obtained by crushing followed by drying. The crushed powder usually gives a zero refusal on a sieve of 60 μm after grinding for about 2 min, which gives it an appreciable fineness. Finally, the homogenizing step consists of mixing the samples obtained in the previous phase in order to obtain a homogeneous, simple, and harmonic approximation. It is done by introducing four catches (of about 250 g each) into a one-liter box and shaking it manually for 2–3 min. The samples are then stored in boxes and numbered for analysis by X-ray fluorescence.

### 3.2 Methods

#### 3.2.1 X-Ray Fluorescence

The X-ray fluorescence technique provides a precise determination of both quantitative and qualitative analysis of chemical oxides (CaO, SiO_2_, Al_2_O_3_, Fe_2_O_3_, MgO, SO_3_, K_2_O, Na_2_O, P_2_O_5_, and F) composition (Khelifi et al., 2017). All treated raw materials were analyzed by the X-Ray florescence ARL9900 spectrometer.

#### 3.2.2 Raw Meal Combination Calculation Tool: Calculation Basis

This part described the calculation tool used to optimize the raw meal combinations; it gives information about the chemical compositions in oxides of optimized raw meals and the mineralogical composition of their corresponding clinkers. The numerical application of this calculation basis is carried on the EXCELL software, which is based on the chemical analysis from XRF of each raw material (ordinary limestone, black marl, flint, etc.) and the burning modules variation (LSF, SIM, and ALM) (Chatterjee, 1983; Mezza et al., 2020).

$$\text{Lime}\;\text{saturation}\;\text{factor:}\;\mathrm{LSF}=\frac{100\times \text{C}}{2,8\times \text{S}+1,18\times \text{A}+0,65\times \text{F}}96\leq LSF\leq 100$$

$$\text{Silica}\;\text{ratio:}\;\text{SIM}=\frac{S}{A+F}2\leq \mathrm{SIM}\leq 3.5$$

$$\text{Alumina}\;\text{ratio:}\;\text{ALM}==\frac{A}{F}0.64\leq \mathrm{ALM}\leq 1.8$$

The LSF, SIM, and ALM ratio are considered the main parameters for raw mix design. So, the steps to be taken to carry out this work are:

• Determination of chemical composition (CaO, SiO_2_, Al_2_O_3_, Fe_2_O_3_, MgO, SO_3_, K_2_O, Na_2_O, P_2_O_5_, and F) in weight percentages of each raw material by means X-ray fluorescence. The raw materials (limestone, marl, etc.) are designated M1, …, Mn (1 < *n* ≤ 4)as presented in Table 3 by means X-ray fluorescence.

**TABLE 3:** Adopted designations used to explain the raw meal (raw mix) calculation program.

Chemical composition	M1	......	Mn	Optimized raw meal	Relative clinker
%SiO_2_	S_M1_	......	S_Mn_	S	S_ck_
%Al_2_O_3_	A_M1_	......	A_Mn_	A	A_ck_
%Fe_2_O_3_	F_M1_	......	F_Mn_	F	F_ck_
%CaO	C_M1_	......	C_Mn_	C	C_ck_
%MgO	M_M1_	......	M_Mn_	M	M_ck_
%SO_3_	${\bar{\text{S}}}_{\text{M}1}$	......	${\bar{\text{S}}}_{\text{Mn}}$	$\bar{\text{S}}$	${\bar{\text{S}}}_{\text{ck}}$
%K_2_O	K_M1_	......	K_Mn_	K	K_ck_
%Na_2_O	N_M1_	......	N_Mn_	N	N_ck_
%P_2_O_5_	P_M1_	......	P_Mn_	P	P_ck_
%F	${\bar{\text{F}}}_{\text{M}1}$	......	${\bar{\text{F}}}_{\text{Mn}}$	$\bar{\text{F}}$	${\bar{\text{F}}}_{\text{ck}}$

S_M1_ is the silica content in M1 (determined from XRF analysis), S is the silica amount in the optimized raw meal combination, and S_ck_ is the SiO_2_ weight percentage in its corresponding clinker.

• Then, with the knowledge of necessary proportions (*x*1, *x*2, …, *xn*) from each raw material (M1…Mn), the chemical composition $\left(\text{S,C,}\ldots \text{,}\bar{\text{F}}\right)$ of optimized raw mix is simply obtained by means the following Eqs 1–10:

S=x1SM1+…+xnSMn (1)

C=x1CM1+…+xnCMn (2)

A=x1AM1+…+xnAMn (3)

F=x1FM1+…+xnFMn (4)

M=x1MM1+…+xnMMn (5)

$$\bar{\text{S}}=x1\bar{\text{S}}M1+\ldots +xn\bar{\text{S}}Mn$$ (6)

K=x1KM1+…+xnKMn (7)

N=x1NM1+…+xnNMn (8)

P=x1PM1+…+xnPMn (9)

$$\bar{\text{F}}=x1\bar{\text{F}}M1+\ldots +xn\bar{\text{F}}Mn$$ (10)

• Using Bogue calculation (Le Saoût et al., 2011; Standard, 2011; Labidi et al., 2019b), the mineralogical composition (C_3_S, C_2_S, C_3_A, and C_4_AF) is determined in order to study the suitability of used raw materials to produce the desired cement type (in our case, SR Portland cement).

##### 3.2.2.1. The Use of Three Raw Materials (*n* = 3) in Optimized Raw Meal Calculation Program

**FIGURE 5:**
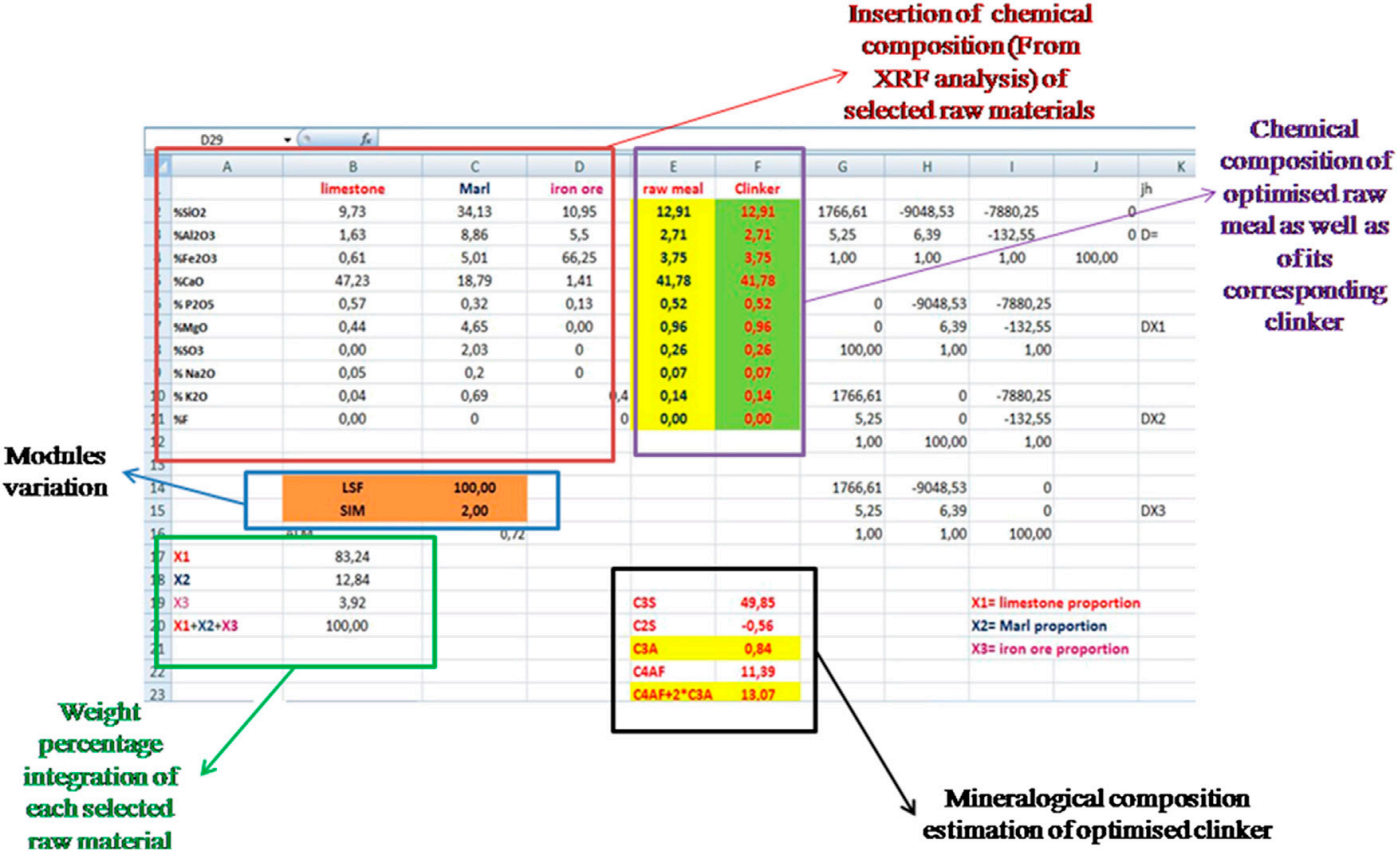
Excel Sheet extracted from the raw meal calculation program which was based on three raw materials.

As mentioned in the previous section, it is necessary to determine the necessary quantities (*x*1, *x*2, and *x*3) from used raw materials, in order to calculate the chemical composition oxides of the optimized raw mix as explained in Figure 5.

So, the calculation of the different percentage integration of raw materials is based on the different stages of the following calculation system:

• Integration of the Eqs 1–4 already elaborated in the previous section, on (I), (II), and (III) formulas:

$$\text{LSF}=\frac{100\times \text{C}}{2.8\times \text{S}+1.18\times \text{A}+0.65\times \text{F}}\;\;\;\;\left(I\right)$$

$$SIM=\frac{S}{A+F}\;\;\;\left(\mathrm{II}\right)$$

x1+x2+x3=100   (III)

The (I), (II), and (III) take new forms as following:

$$a_{1}\times x1+a_{2}\times x2+a_{3}\times x3=0\;\;\left(\mathrm{I-1}\right)$$

$$a_{4}\times x1+a_{5}\times x2+a_{6}\times x3=0\;\;\;\left(\mathrm{II-1}\right)$$

x1+x2+x3=100   (III-1)

Since

$$a_{1}=100\times C_{M1}-LSF\times \left(2.8\times S_{M1}+1.18\times A_{M1}+0.65\times F_{M1}\right);$$

$$a_{2}=100\times C_{M2}-LSF\times \left(2.8\times S_{M2}+1.18\times A_{M2}+0.65\times F_{M2}\right);$$

$$et\;a_{3}=100\times C_{M3}-LSF\times \left(2.8\times S_{M3}+1.18\times A_{M3}+0.65\times F_{M3}\right);$$

$$a_{4}=S_{M1}-SIM\times \left(A_{M1}+F_{M1}\right);$$

$$a_{5}=S_{M2}-SIM\times \left(A_{M2}+F_{M2}\right);$$

$$a_{6}=S_{M3}-SIM\times \left(A_{M3}+F_{M3}\right).$$

• Resolution of the computing system includes (I-1), (II-1), and (III-1) by means of Cramer mathematical method as present:

$$\left(\begin{array}{ccc} a1 & a2 & a3\\ a4 & a5 & a6\\ 1 & 1 & 1 \end{array}\right)\times \left(\begin{array}{c} X1\\ \begin{array}{c} X2\\ X3 \end{array} \end{array}\right)=\left(\begin{array}{c} 0\\ 0\\ 100 \end{array}\right)$$

$$A1=\det\left|\begin{array}{ccc} \text{a}1 & \text{a}2 & \text{a}3\\ \text{a}4 & \text{a}5 & \text{a}6\\ 1 & 1 & 1 \end{array}\right|=a1\times \left(a5\times 1-a6\times 1\right)-a4\times \left(a2\times 1-a3\times 1\right)+1\times \left(a2\times a6-a5\times a3\right);$$

$$A2=\det\left|\begin{array}{ccc} 0 & a2 & a3\\ 0 & a5 & a6\\ 100 & 1 & 1 \end{array}\right|=100\left(a2\times a6-a5\times a3\right);$$

$$A3=\det\left|\begin{array}{ccc} a1 & 0 & a3\\ a4 & 0 & a6\\ 1 & 100 & 1 \end{array}\right|=a1\times \left(-a6\times 100\right)-a4\times \left(-a3\times 100\right);$$

$$A4=\det\left|\begin{array}{ccc} a1 & a2 & 0\\ a4 & a5 & 0\\ 1 & 1 & 100 \end{array}\right|=a1\times \left(a5\times 100\right)-a4\times \left(a2\times 100\right);$$

And the x1 = A2/A1, x2 = A3/A1and x3 = A4/A1.

• Once the *x*1, *x*2, and *x*3 rates are calculated, the chemical composition of the optimized raw meal is determined Eq 1–10, which are used subsequently to estimate the mineralogical composition of its corresponding clinker (see Figure 5).

##### 3.2.2.2 The Use of Four Raw Materials (*n* = *4*) in Optimized Raw Meal Calculation Program

The same methodology is used as in the previous part:

• Integration of the Eqs 1–4 already elaborated in the previous section, on (I), (II), (III), and (IV) formulas:

$$\text{LSF}=\frac{100\times \text{C}}{2.8\times \text{S}+1.18\times \text{A}+0.65\times \text{F}}\;\;\;\left(I\right)$$

$$SIM=\frac{S}{A+F}\;\;\;\left(\mathrm{II}\right)$$

$$ALM==\frac{A}{F}\;\;\;\left(\mathrm{III}\right)$$

x1+x2+x3+x4=100   (IV)

The (I), (II), (III), and (IV) equations take new forms as follows:

b1×x1+b2×x2+b3×x3+b4×x4=0  (I-2)

b5×x1+b6×x2+b7×x3+b8×x4=0  (II-2);

c1×x1+c2×x2+c3×x3+c4×x4=0  (III-2);

x1+x2+x3+x4=100  (IV-2);

Since

$$b_{1}=100\times C_{M1}-LSF\times \left(2.8\times S_{M1}+1.18\times A_{M1}+0.65\times F_{M1}\right);$$

$$b_{2}=100\times C_{M2}-LSF\times \left(2.8\times S_{M2}+1.18\times A_{M2}+0.65\times F_{M2}\right);$$

$$b_{3}=100\times C_{M3}-LSF\times \left(2.8\times S_{M3}+1.18\times A_{M3}+0.65\times F_{M3}\right);$$

$$b_{4}=100\times C_{M4}-LSF\times \left(2.8\times S_{M4}+1.18\times A_{M4}+0.65\times F_{M4}\right);$$

$$b_{5}=S_{M1}-SIM\times \left(A_{M1}+F_{M1}\right);$$

$$b_{6}=S_{M2}-SIM\times \left(A_{M2}+F_{M2}\right);$$

$$b_{7}=S_{M3}-SIM\times \left(A_{M3}+F_{M3}\right);$$

$$b_{8}=S_{M4}-SIM\times \left(A_{M4}+F_{M4}\right);$$

$$c_{1}=A_{M1}-\left(ALM\times F_{M1}\right);$$

$$c_{2}=A_{M2}-\left(ALM\times F_{M2}\right);$$

$$c_{3}=A_{M3}-\left(ALM\times F_{M3}\right);$$

$$c_{4}=A_{M4}-\left(ALM\times F_{M4}\right);$$

• Resolution of the computing system includes (I-2), (II-2), (III-2), and (IV-2) equations by means of the Cramer mathematical method as follows:

$$\left(\begin{array}{cccc} b1 & b2 & b3 & b4\\ b5 & b6 & b7 & b8\\ c1 & c2 & c3 & c4\\ 1 & 1 & 1 & 1 \end{array}\right)\times \left(\begin{array}{c} X1\\ \begin{array}{c} X2\\ X3\\ x4 \end{array} \end{array}\right)=\left(\begin{array}{c} 0\\ 0\\ 0\\ 100 \end{array}\right)$$

$$\begin{array}{l} B1=\det\left|\begin{array}{cccc} b1 & b2 & b3 & b4\\ b5 & b6 & b7 & b8\\ c1 & c2 & c3 & c4\\ 1 & 1 & 1 & 1 \end{array}\right|=b1\times \det\left|\begin{array}{ccc} b6 & b7 & b8\\ c2 & c3 & c4\\ 1 & 1 & 1 \end{array}\right|-b5\times \\ det\left|\begin{array}{ccc} b2 & b3 & b4\\ c2 & c3 & c4\\ 1 & 1 & 1 \end{array}\right|+c1\times \det\left|\begin{array}{ccc} b2 & b3 & b4\\ b6 & b7 & b8\\ 1 & 1 & 1 \end{array}\right|-\det\left|\begin{array}{ccc} b2 & b3 & b4\\ b6 & b7 & b8\\ c2 & c3 & c4 \end{array}\right|\\ =b1\times \left[b6\times \left(c3-c4\right)-c2\times \left(b7-b8\right)+\left(b7\times c4-c3\times b8\right)\right]-b5\times \left[b2\times \left(c3-c4\right)-c2\times \left(b3-b4\right)+\left(b3\times c4-c3\times b4\right)\right]+\\ c1\times \left[b2\times \left(b7-b8\right)-b6\times \left(b3-b4\right)+\left(b3\times b80b7\times b8\right)\right]-\left[b2\times \left(b7\times c4-c3\times b8\right)-b6\times \left(b3\times c4-c3\times b4\right)+c2\times \left(b3\times b8-b7\times b4\right)\right] \end{array}$$

$$B2=\det\left|\begin{array}{cccc} 0 & b2 & b3 & b4\\ 0 & b6 & b7 & b8\\ 0 & c2 & c3 & c4\\ 100 & 1 & 1 & 1 \end{array}\right|=-100\times \det\left|\begin{array}{ccc} b2 & b3 & b4\\ b6 & b7 & b8\\ c2 & c3 & c4 \end{array}\right|=100\times b2\times \left(b7\times c4=c3\times b8\right)-b6\times \left(b3\times c4-c3\times b4\right)+c2\times \left(b3\times b8-b7\times b4\right)$$

$$\begin{array}{l} B3=\det\left|\begin{array}{cccc} b1 & 0 & b3 & b4\\ b5 & 0 & b7 & b8\\ c1 & 0 & c3 & c4\\ 1 & 100 & 1 & 1 \end{array}\right|=b1\times \det\left|\begin{array}{ccc} 0 & b7 & b8\\ 0 & c3 & c4\\ 100 & 1 & 1 \end{array}\right|-b5\times \det\left|\begin{array}{ccc} 0 & b3 & b4\\ 0 & c3 & c4\\ 100 & 1 & 1 \end{array}\right|+c1\times \det\left|\begin{array}{ccc} 0 & b3 & b4\\ 0 & b7 & b8\\ 100 & 1 & 1 \end{array}\right|-\det\left|\begin{array}{ccc} 0 & b3 & b4\\ 0 & b7 & b8\\ 0 & c3 & c4 \end{array}\right|\\ =b1\times 100\times \left(b7\times c4-b8vc3\right)-b5\times 100\times \left(b3\times c4-c3\times b4\right)+c1\times 100\times \left(b3\times b8-b7\times b4\right) \end{array}$$

$$\begin{array}{l} B4=\det\left|\begin{array}{cccc} b1 & b2 & 1 & b4\\ b5 & b6 & b7 & b8\\ c1 & c2 & c3 & c4\\ 1 & 1 & 100 & 1 \end{array}\right|=b1\times \det\left|\begin{array}{ccc} b6 & 0 & b8\\ c2 & 0 & c4\\ 1 & 100 & 1 \end{array}\right|-b5\times \det\left|\begin{array}{ccc} b2 & 0 & b4\\ c2 & 0 & b4\\ 1 & 100 & 1 \end{array}\right|+c1\times \det\left|\begin{array}{ccc} b2 & 0 & b4\\ b6 & 0 & b8\\ 1 & 100 & 1 \end{array}\right|-\det\left|\begin{array}{ccc} b2 & 0 & b4\\ b6 & 0 & b8\\ c2 & 0 & c4 \end{array}\right|\\ =b1\times \left[b6\times \left(-100\times c4\right)-c2\times \left(-100\times b8\right)\right]-b5\times \left[b2\times \left(-100\times c4\right)-c2\times \left(-100\times b4\right)\right]+c1\times \left[b2\times \left(-100\times b8\right)-b6\times \left(-100\times b4\right)\right] \end{array}$$

$$\begin{array}{l} B5=\det\left|\begin{array}{cccc} b1 & b2 & b3 & 0\\ b5 & b6 & b7 & 0\\ c1 & c2 & c3 & 0\\ 1 & 1 & 1 & 100 \end{array}\right|=b1\times \det\left|\begin{array}{ccc} b6 & b7 & 0\\ c2 & c3 & 0\\ 1 & 1 & 100 \end{array}\right|-b5\times \det\left|\begin{array}{ccc} b2 & b3 & 0\\ c2 & c3 & 0\\ 1 & 1 & 100 \end{array}\right|+c1\times \det\left|\begin{array}{ccc} b2 & b3 & 0\\ b6 & b7 & 0\\ 1 & 1 & 100 \end{array}\right|-\det\left|\begin{array}{ccc} b2 & b3 & 0\\ b6 & b7 & 0\\ c2 & c3 & 0 \end{array}\right|\\ =b1\times \left[b6\times \left(c3\times 100\right)-c2\times \left(100\times b7\right)\right]-b5\times \left[b2\times \left(-100\times c3\right)-c2\times \left(b3\times 100\right)\right]+c1\times \left[b2\times \left(b7\times 100\right)-b6\times \left(100\times b3\right)\right] \end{array}$$

And finally, the rates of each raw material are:

x1=B2/B1,x2=B3/B1,x3=B4/B1et x4=B5/B1

• Once the x1, x2, x3, and x4 rates are calculated, the chemical composition of the optimized raw meal is determined Eqs 1–10, which are used subsequently to estimate the mineralogical composition of its corresponding clinker.

## 4 Results and Discussions

### 4.1 XRF Analysis

The chemical composition analysis, determined by XRF, of different raw materials was presented in Table 4, as well as the quantities of carbonate calcium.

**TABLE 4:** Chemical analysis of collected raw materials (in weight percentages).

	% SiO_2_	%Al_2_O_3_	%Fe_2_O_3_	%CaO	%MgO	%SO_3_	%K_2_O	%Na_2_O	%P_2_O_5_	%F	%CaCO_3_
Ordinary limestone	9.73	1.63	0.61	47.23	0.44	0.00	0.04	0.05	0.57	—	83.33
Grey marl	28.46	9.20	4.35	23.79	2.57	0.75	0.47	0.07	0.27	—	42.48
Black marl	34.13	8.86	5.01	18.79	4.65	2.08	0.69	0.20	0.32	—	33.55
Iron ore	10.95	5.50	66.25	1.41	0.00	0.00	0.40	0.00	0.13	—	—
Siliceous limestone	15.17	1.89	0.73	44.57	0.64	0.00	0.06	0.05	0.69	—	79.60
Yellow marl	21.67	6.52	5.42	35.10	1.72	0.88	0.54	0.09	0.42	—	62.69
Black limestone	9.60	2.10	0.6	45.86	0.51	0.64	0.06	0.04	0.37	—	81.36
Flint	72.45	0.78	0.91	15.37	2.13	0.83	0.00	0.09	0.31	—	27.44
NFA	5.60	0.65	0.35	48.50	0.60	3.50	—	—	28.00	3.30	—

As shown in Table 4, ordinary limestone is characterized by 47.23% of CaO content and close to 10% of silica oxide content. This limestone type is used as the foundation in the SCB factory’s raw meal preparation because it produces high-quality Portland clinker that meets the company’s technical and commercial requirements. However, the siliceous limestone presents a high SiO_2_ amount, about 15.17%. That is why this raw material is known as siliceous limestone. In addition, the black limestone has a similar chemical composition to ordinary limestone.

Comparing the marl samples, the black marl is rich in magnesium oxide (4.65%), while the yellow marl has the lowest MgO content 1.72% and the highest Fe_2_O_3_ amount at about 5.42%. The grey marl is considered an aluminous marl since it contains a high content of alumina (Al_2_O_3_). Concerning the flint, which is considered a very hard sedimentary rock, it is characterized by a very high content of SiO_2_ of 72.45% and low quantities of CaO, Al_2_O_3_, and Fe_2_O_3_. These elements are considered impurities in this rock, which belongs to the family of carbonate rocks (Paul and Kouamé, 2022).

**FIGURE 6:**
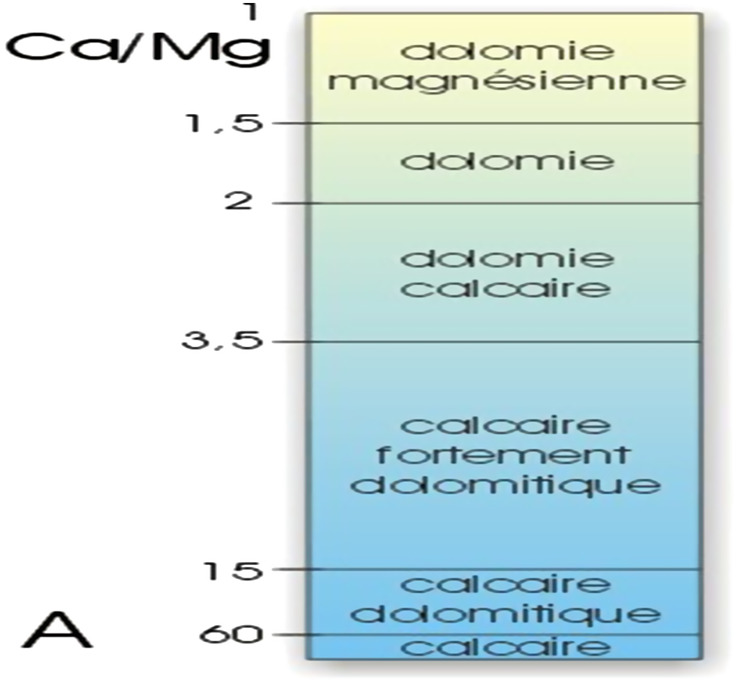
Classification of carbonate rocks according to the Ca/Mg ratio (Boulvain, 2014).

According to the limestone classification scale given in Figure 6, which is based on the Ca/Mg ratio (Boulvain, 2014; Chilingar, 1956; Zeyen et al., 2017; Taha1 and Abdullah2, 2020), the collected limestone samples, as shown in Table 5, are considered pure limestone, poor in dolomite phase [CaMg(CO_3_)_2_]. This means that most of the CaO quantity exists in these samples as CaCO_3_ phase. This constatation is very important to be sure that the CO_2_ emission source is only from CaCO_3_ burning.

**TABLE 5:** Ca/Mg values of collected limestone sample**s.**

	Ordinary limestone	Siliceous limestone	Black limestone
Ca/Mg	107.34	69.64	89.92

The chemical analysis of natural fluorapatite “Ca_5_(PO_4_)_3_F”shows that this raw material presents an important source of CaO (48.50%), which thereafter provides the necessary quantity of lime for the formation of the principal mineralogical phases of Portland cement (C_3_S, C_2_S, C_3_A, and C_4_AF). While the NAF contains an important amount of P_2_O_5_ (28%), phosphorus is considered a detrimental element for both the clinker mineralogy and the physical-mechanical properties of the cement during hydration (Hökfors et al., 2015; Ismail et al., 2015). Moreover, the presence of CaF_2_ (Da et al., 2022) in the NAF structure is considered a key strength since the existence of fluorite in cement raw meal improves the burnability process and lowers the clinkering temperature, which favors the C_3_S formation and reduces the free lime content (Yamashita and Tanaka, 2012; Boughanmi et al., 2018a; Xie et al., 2021).

The natural fluorapatite sample used in this study is already used in published research work (Boughanmi et al., 2018b) about the synthesis of Portland clinker by means of NAF as a raw material at laboratory scale (by using pure commercially available limestone, silica, alumina, and iron oxide as raw materials to prepare raw meals). This work aims to study the influence of the replacement of the proportion of limestone in the raw meals of laboratory made by NAF (0%–15%). It was found (Boughanmi et al., 2018b) that the detrimental impact of phosphorus on the transformation of C_2_S and lime in C_3_S, which is generally remarked for lower amounts, becomes efficient but remains suitable for up to 8% natural fluorapatite in the raw meal. This shows that a beneficial impact of fluorine counterbalances the negative effect of phosphorous. Cements obtained from up to 8% natural fluorapatite incorporation still present acceptable properties (Boughanmi et al., 2018b).

### 4.2 Sulfate-Resisting Portland Cement Raw Meal Optimization

#### 4.2.1 Feasibility Study of SR Portland Cement Raw Meal Preparation

In general, Portland cement manufacturing using only limestone and marl is not usually sufficient to achieve the suitable raw mix in question. Therefore, the use of corrective elements such as iron ore (Fe_2_O_3_ source), sand (SiO_2_ source), or bauxite (Al_2_O_3_ source) is essential in order to balance and correct the raw meal combinations (Michaux et al., 1990; Kurdowski, 2014).

The SCB plant’s raw mix manufacturing uses iron ore as a corrective element in addition to ordinary limestone and gray and black marls. So, based on the raw meal calculation program (the use case three raw materials), the LSF and SIM ratios varied in the range of 96–100 and 2–2.5, respectively, in order to check the suitability of optimized SR raw meal (SR RM) to give a sulfate-resisting Portland clinker (C_3_A ≤ 3% and 2C_3_A + C_4_AF ≤ 20%).

Table 6 presents the calculation results of different SR raw meal combinations; the SR RM10 is considered the only optimized raw meal. It can provide an approximate SR Portland cement (C_3_A ≤ 3), but the second requirement “2C_3_A + C_4_AF ≤ 20%” seems not to be met.

**TABLE 6:** SR Portland raw meal combinations by using three raw materials used in ordinary cement production in the SCB factory.

Combinations	LSF	SIM	%*x*_1_ = integration rate of ordinary limestone	%*x*2 = integration rate of marl	%*x*3 = integration rate of iron ore	%C_3_A	2 × %C_3_A + %C_4_AF	ALM
SR RM1	100	2.5	81.90	16.90	1.71	5.53	22.17	1.21
SR RM 2	99	2.5	81.47	16.85	1.69	5.64	22.41	1.22
SR RM 3	98	2.5	81.03	17.31	1.67	5.75	22.65	1.23
SR RM 4	97	2.5	80.58	17.77	1.65	5.87	22.90	1.24
SR RM 5	96	2.5	80.12	18.25	1.63	5.98	23.14	1.25
SR RM 6	100	2.1	81.88	14.92	3.20	2.94	21.58	0.86
SR RM 7	100	2.2	81.89	15.33	2.78	3.66	21.46	0.94
SR RM 8	100	2.3	81.89	15.71	2.40	4.33	21.72	1.02
SR RM 9	100	2.4	81.90	16.06	2.04	4.95	21.95	1.11
SR RM 10	100	2	81.88	14.47	3.65	2.16	20.88	0.79
SR RM 11	99	2	81.49	14.97	3.64	2.25	21.11	0.79
SR RM 12	98	2	81.07	15.36	3.36	2.34	21.34	0.80
SR RM 13	97	2	80.57	15.80	3.63	2.43	21.57	0.81
SR RM 14	96	2	80.12	16.26	3.62	2.52	21.81	0.81

Marl = 50% grey marl+50% black marl.

The SR RM10combination contains a high iron ore incorporation percentage (3.65%), which explains the decrease in aluminate content (C_3_A) and consequently the increase of 2C_3_A + C_4_AF.This behaviour can favor the formation of C_2_F phase during the clinkering process. However, the increase of iron oxide in raw meal affects the cement kiln process. In fact, the high amount of Fe_2_O_3_ induces the decrease of the clinker liquid phase “L.P” viscosity during the burning process. In this case, the liquid phase becomes very fluid and there is a high risk of ring formation in the kiln, which causes the blockage of the burning lining (Kumar, 2013; Belgacem et al., 2016; Sengupta, 2020). So, it is imperative to optimize a good raw meal (especially if it is not doped with iron ore) to obtain SR Portland cement with a low amount of aluminate phase (C_3_A) and to prevent poor operation of the cement kiln.

The LSF = 100 and SIM = 2 are considered acceptable values since they are situated in a suitable range of values for Portland cement manufacturing.

The C_2_S content of the SR RM 10 combination is negative (−0.44%), indicating that the relative clinker is poor in belite phase. Subsequently, the clinker grinding becomes easier since the alite amount increases and ameliorates the clinker grindability (Unland, 2001).

To improve the quality of optimized SR raw meal without increasing the iron ore incorporation, a replacement of marl by only black marl was necessary, in order to increase the silica content and maintain the 2C_3_A + C_4_AF at less than 20%. This study (Table 7) provides the possibility to obtain a SR Portland raw meal (SR RM 16) required to meet the SR Portland cement requirements (C_3_A ≤ 3 and 2C_3_A + C_4_AF ≤ 20%) (NT, 47.26, 1999; En, 2001).

**TABLE 7:** SR Portland raw meal combinations by using three raw materials (substituting the marl mix with black marl).

Combinations	LSF	SIM	%*x*_1_ = integration rate of ordinary limestone	%*x*2 = integration rate of black marl	%*x*3 = integration rate of iron ore	%C_3_A	2 × %C_3_A + %C_4_AF	ALM
SR RM 15	100	2	83.25	12.85	3.89	1.27	19.78	0.72
SR RM 16	99	2	82.87	13.24	3.90	1.34	19.97	0.73
SR RM 17	98	2	82.47	13.63	3.90	1.40	20.17	0.73
SR RM 18	97	2	82.07	14.03	3.90	1.46	20.37	0.73
SR RM 19	96	2	81.66	14.43	3.91	1.56	20.57	0.74

So incorporating siliceous marl (black marl) can be a solution to produce an SR Portland clinker. Whereas, the manufacturing of this combination (SR RM 16) on an industrial scale is difficult since the use of ALM value of about 0.73 makes the burning process hard (Spence, 1980).

#### 4.2.2 SR Portland Raw Meal Optimization With Correction

In this part, the calculation of optimized raw meals is based on the variation of all three burning modules (LSF, SIM, and ALM) and the integration of the fourth raw material (flint and siliceous limestone) being extracted. The choice of these materials is related to the important content of silica, as shown in Table 4.

Based on Bogue equations (C_3_A = 2.650 × %Al_2_O_3_−1.692 × %Fe_2_O_3_ and C_4_AF = 3.043 × %Fe_2_O_3_), if C_3_A was fixed at 3%, the ALM modulus would be taken to a value of about 0.91. This ALM value will be used in raw meals’ optimization since it offers a high probability of manufacturing SR Portland cement and guarantees the stability of the burning lining (kiln) of the cement manufacturing process.

##### 4.2.2.1 SR Portland Raw Meal Correction With Flint and Siliceous Limestone

This study of raw meal optimization (calculation program of four raw materials) is based on the incorporation (%x4) of a fourth raw material (flint or siliceous limestone) in addition to limestone (%x1), marl (%x2), and iron ore (%x3). The choice of these corrective materials is based on the high silica content they contain (see Table 4).

• Flint integration

The calculation results of optimized raw meal combinations with flint correction are as listed below:

- SR RM 20: LSF = 100, SIM = 2, ALM = 0.91; %*x*1 = 80.71, %*x*2 = 17.75, %*x*3 = 2.73; %*x*4 = −0.91; %C_3_A = 3.59 and 2 × %C_3_A + %C_4_AF = 22.37.
- SR RM 21: LSF = 100, SIM = 2.3, ALM = 0.91; %*x*1 = 82.91, %*x*2 = 13.87, %*x*3 = 2.42, %*x*4 = 0.78; % C_3_A = 3.24 and 2 × %C_3_A + %C_4_AF = 20.15.
- SR RM 22: LSF = 100, SIM = 2.5, ALM = 0.91; %*x*1 = 84.17, %*x*2 = 11.69, %*x*3 = 2.25, %*x*4 = 1.89; %C_3_A = 3.04 and 2 × %C_3_A + %C_4_AF = 18.90.
- SR RM 23: LSF = 100, SIM = 3, ALM = 0.91; %*x*1 = 86.66, %*x*2 = 7.32, %*x*3 = 1.90, %*x*4 = 4.12; %C_3_A = 2.63 and 2 × %C_3_A + %C_4_AF = 16.36.
- SR RM 24: LSF = 99, SIM = 3, ALM = 0.91; %*x*1 = 86.32, %*x*2 = 7.55, %*x*3 = 1.92, %*x*4 = 4.12; %C_3_A = 2.65 and 2 × %C_3_A + %C_4_AF = 16.48.
- SR RM 25: LSF = 98, SIM = 3, ALM = 0.91; %*x*1 = 85.97, %*x*2 = 7.79, %*x*3 = 1.93, %*x*4 = 4.31; %C_3_A = 2.66 and 2 × %C_3_A + %C_4_AF = 16.59
- SR RM 26: LSF = 97, SIM = 3, ALM = 0.91; %*x*1 = 85.61, %*x*2 = 8.04, %*x*3 = 1.95, %*x*4 = 4.40; %C_3_A = 2.68 and 2 × %C_3_A + %C_4_AF = 16.70.
- SR RM 27: LSF = 96, SIM = 3, ALM = 0.91; %*x*1 = 85.25, %*x*2 = 8.28, %*x*3 = 1.97, %*x*4 = 4.50; %C_3_A = 3.70 and 2 × %C_3_A + %C_4_AF = 16.52.

The flint incorporation improved the specific mineralogical composition of the optimized SR Portland clinker. So, in this case, the SR Portland cement manufacturing cement is possible from the flint incorporation, satisfying the appropriate integration level of flint. The raw mix “SR RM 22” is the best optimized combination since it presents the optimum conditions of SR Portland cement.

• Siliceous limestone integration

The siliceous limestone is characterized by a silica content of about 15% and a hardness of three according to the Mohs hardness scale (Gibson et al., 2018). This rock is easy to grind because its humidity is on order of 5%. The calculation results of optimized raw meal combinations with siliceous limestone correction are as listed below:

- SR RM 27: LSF = 100, SIM = 2, ALM = 0.91; %*x*1 = 80.71, %*x*2 = 17.75, %*x*3 = 2.73, %*x*4 = −0.91; %C_3_A = 3.59 and 2 × %C_3_A + %C_4_AF = 22.37.
- SR RM 28: LSF = 100, SIM = 2.5, ALM = 0.91; %*x*1 = 60.05, %*x*2 = 10.54, %*x*3 = 2.40, %*x*4 = 27.01; %C_3_A = 3.05 and 2 × %C_3_A + %C_4_AF = 18.97.
- SR RM 29: LSF = 100, SIM = 2.6, ALM = 0.91; %*x*1 = 54.20, %*x*2 = 6.24, %*x*3 = 2.23, %*x*4 = 34.19; %C_3_A = 2.96 and 2 × %C_3_A + %C_4_AF = 18.42.
- SR RM 30: LSF = 100, SIM = 3, ALM = 0.91; %*x*1 = 33.98, %*x*2 = 4.77, %*x*3 = 2.23, %*x*4 = 59.02; %C_3_A = 2.65 and 2 × %C_3_A + %C_4_AF = 16.50.
- SR RM 31: LSF = 99, SIM = 3, ALM = 0.91; %*x*1 = 35.45, %*x*2 = 4.95, %*x*3 = 2.26, %*x*4 = 60.34; %C_3_A = 2.67 and 2 × %C_3_A + %C_4_AF = 16.61.
- SR RM 32: LSF = 98, SIM = 3, ALM = 0.91; %*x*1 = 30.90, %*x*2 = 5.13, %*x*3 = 2.28, %*x*4 = 61.69; %C_3_A = 2.69 and 2 × %C_3_A + %C_4_AF = 16.73.
- SR RM 33: LSF = 97, SIM = 3, ALM = 0.91; %*x*1 = 29.32, %*x*2 = 5.32, %*x*3 = 2.30, %*x*4 = 63.06; %C_3_A = 2.71 and 2 × %C_3_A + %C_4_AF = 16.85.
- SR RM 34: LSF = 96, SIM = 3, ALM = 0.91; %*x*1 = 27.72, %*x*2 = 5.51, %*x*3 = 2.33, %*x*4 = 64.45; %C_3_A = 2.73 and 2 × %C_3_A + %C_4_AF = 16.97.

The results of the calculation show that SR Portland cement manufacturing with this limestone type is possible and that its integration rates vary from 27% to 64%. Moreover, the burning module values in this studied case are considered suitable in the rage of Portland cement manufacturing without undesirable effects on the operation of the cement factory.

It is possible to prepare a SR raw meal using flint, but this latter can eventually induce a problem in the raw materials grinding operation. Since flint is a very hard rock (rich in quartz), its hardness was estimated to be seven on the mohs hardness scale (Bennett et al., 1989; Beuker, 2012). Therefore, the manufacturing of SR clinker using flint requires enormous energy during the operation of raw mix milling and subsequently high and costly energy consumption.

So the only way to exploit the flint deposit is to mix a low quantity of this material with a high rate of siliceous limestone, since the latter is a brittle material and improves the flint grindability. So the fourth material requires a combination of “95% siliceous limestone + 5% flint.”

The calculation results of optimized raw meal combinations with combined siliceous limestone and flint correction are as listed below:

- SR RM 35: LSF = 100, SIM = 3, ALM = 0.91; %*x*1 = 56.63, %*x*2 = 5.86, % *x*3 = 2.09, %*x*4 = 35.42; %C_3_A = 2.65 and 2 × %C_3_A + %C_4_AF = 16.44.
- SR RM 36: LSF = 99, SIM = 3, ALM = 0.91; %*x*1 = 55.62, %*x*2 = 6.07, %*x*3 = 2.11, %*x*4 = 36.21; %C_3_A = 2.66 and 2 × %C_3_A + %C_4_AF = 16.55.
- SR RM 37: LSF = 98, SIM = 3, ALM = 0.91; %*x*1 = 54.57, %*x*2 = 6.28, %*x*3 = 2.13, %*x*4 = 37.02; %C_3_A = 2.68 and 2 × %C_3_A + %C_4_AF = 16.67.
- SR RM 38: LSF = 97, SIM = 3, ALM = 0.91; %*x*1 = 53.52, %*x*2 = 6.49, %*x*3 = 2.15, %*x*4 = 37.84; %C_3_A = 2.70 and 2 × %C_3_A + %C_4_AF = 16.79
- SR RM 39: LSF = 96, SIM = 3, ALM = 0.91; %*x*1 = 52.46, %*x*2 = 6.49, %*x*3 = 2.17, %*x*4 = 38.67; % C_3_A = 2.72 and 2 × %C_3_A + %C_4_AF = 16.90.

From the calculation results of the different possible formulas of SR raw mix using the mix integration between flint and siliceous limestone, it is clear that this kind of combination presents a good solution to produce the SR Portland cement required to standard specification, since the used values of burning modules are strictly in the ranges of the well performing cement kiln: 96 ≤ LSF ≤ 100, SIM = 3 and ALM = 0.91.

#### 4.2.3. Natural Fluorapatite Integration in SR Raw Meals

According to previous research works, the primary goal of using natural fluorapatite in this study is to:

- Improve the quality of SR Portland cement (C_3_A ≤ 3% and 2C_3_A + C_4_AF ≤ 20%.)
- Ensure the stability of the cement manufacturing process.
- Reduce the CO_2_ emissions in the cement industry.

So, this was studied in the present paper by following the effect of the replacement of a part of the ordinary limestone in the raw materials (SRRM 10, SRRM 16, SRRM 23, SRRM 30, and SRRM 35) with natural fluorapatite (0%–15%)on their mineralogical composition (mainly C_3_A) and the liquid phase percentage formation. The latter plays a critical role in the clinkering development and the burning stability process, as explained in Section 2.

**FIGURE 7:**
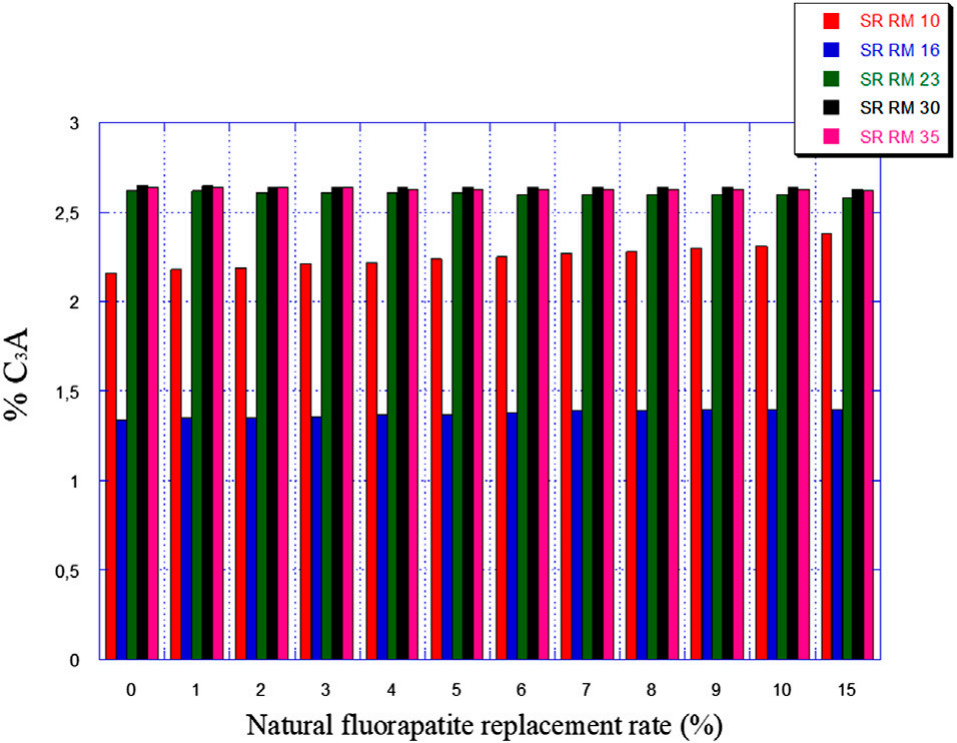
Expected C_3_A amounts variation in function of the natural fluorapatite replacement.

**FIGURE 8:**
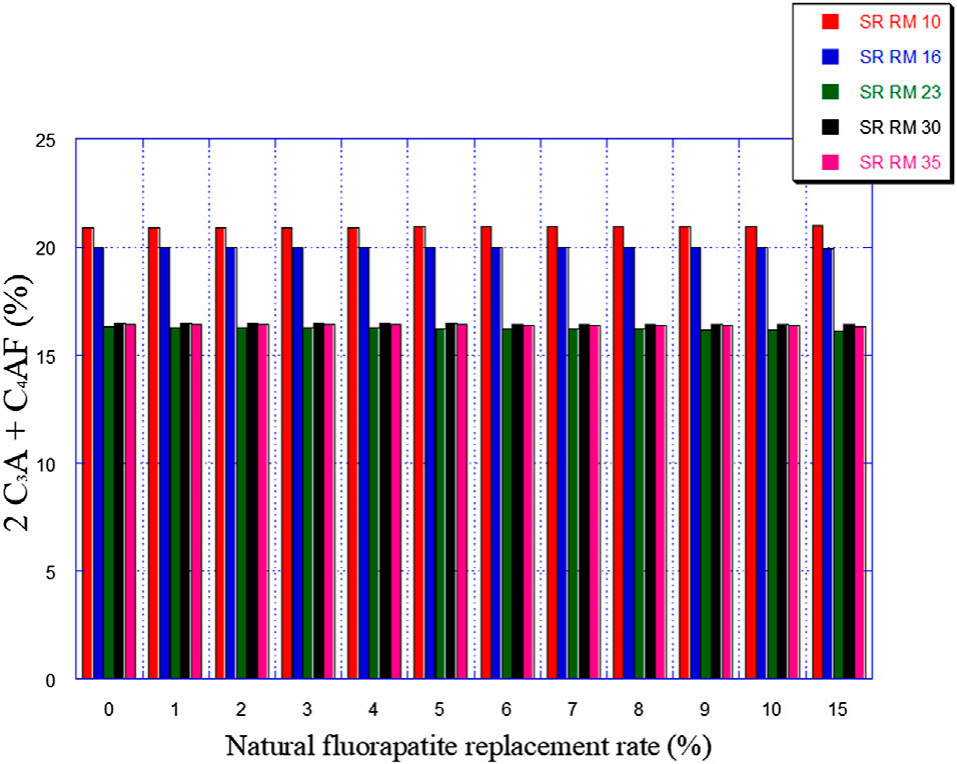
Expected 2C_3_A + C_4_AF amounts variation in function of the natural fluorapatite replacement.

Figures 7, 8 illustrate the expected variation in C_3_A and 2C_3_A + C_4_AF amounts as a function of natural fluorapatite replacement.

**FIGURE 9:**
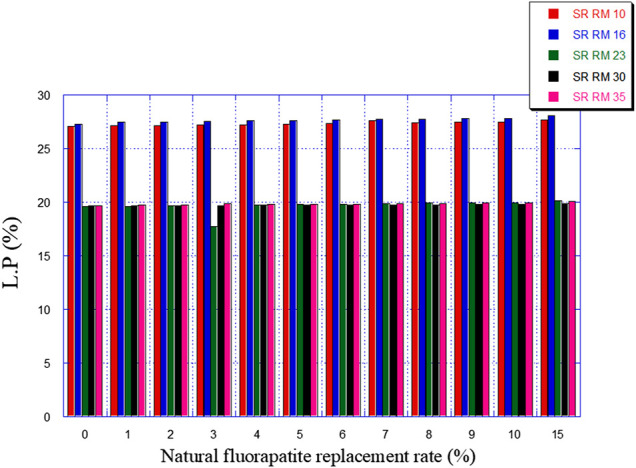
Predicted liquid phase (L.P) amounts in function of the natural fluorapatite replacement.

**FIGURE 10:**
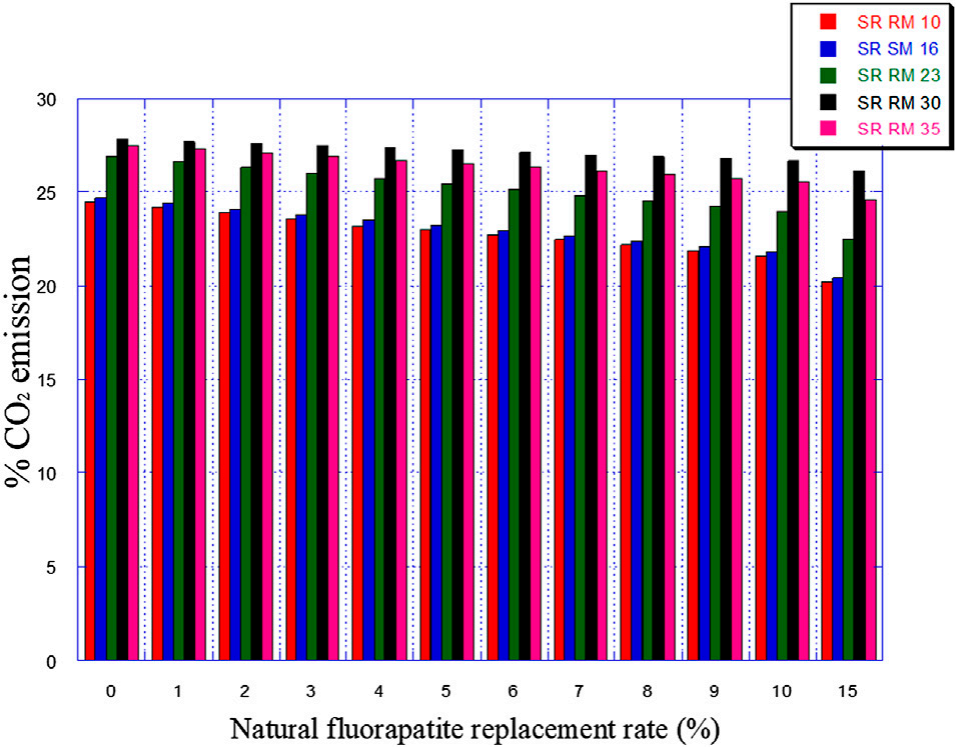
Expected CO_2_ emissions in function of the natural fluorapatite replacement.

The natural fluorapatite replacement on raw meals (SR RM 10, SR RM 16, SR RM 23, and SR RM 35) showed that:

• The natural fluorapatite substitution promotes the liquid phase formation (see Figure 9). The calculated LP values seem satisfactory since these estimated melts belong to the range of the coating stability of the cement kiln refractory lining. (Aye and Oguchi, 2011). Liquid phase prediction is considered an important and fundamental task for a cement manufacturer in order to ensure the good functioning of the cement production line (burning phase).
• All the estimated raw mix combinations are in agreement with the SR Portland cement normative requirements (C_3_A ≤ 3% et 2C_3_A + C_4_AF ≤ 20%), as indicated in Figures 7, 8.
• The natural fluorapatite replacement reduces the iron oxide amount in the SR Portland cement raw meals (Figure 8); this behavior decreases the polymorphic conversion probability of the belite phase from β C_2_S to γ C_2_S during the clinkering process (Odigure, 1996; Odigure, 1999; Labidi et al., 2019b)and the γ C_2_S mineral formation has a lower reactivity with water (de Noirfontaine, 2000) and consequently affects the mechanical and physical properties of cement (Odigure, 1999).
• An important reduction of greenhouse gas emissions “%CO_2_” (see Figure 10)

Moreover, the fluorite (CaF_2_) in natural fluorapatite, as known as a melting agent, improves the burning phase by increasing the liquid phase formation and decreasing the clinkering temperature (Johansen and Christensen, 1979; Klemm et al., 1979; Odler and Schmidt, 1980; Raina and Janakiraman, 1998; Altun, 1999b; Yamashita and Tanaka, 2012).

## 5 Conclusion

In the present work, different cases were studied to estimate the raw meal combinations suitable for sulfate-resisting Portland cement manufacturing.

The raw materials (ordinary limestone, black marl, grey marl, and iron ore) used for the ordinary Portland cement production in the SCB cement plant were not sufficient to produce SR raw meals. Thus, the materials abandoned in the SCB quarries (siliceous limestone and flint) were a solution to preparing a SR raw meal.

The results show that the integration of “5% flint with 95% siliceous limestone” mixture is considered a suitable combination for SR clinker while maintaining the same raw materials, burning modules (LSF = 100, SIM = 3, and ALM = 0. 91), and the same manufacturing process. This integration allows the cement factory (SCB plant) to take care of its own integration of all abandoned fronts in the preparation of the raw mix. Furthermore, the use of these abandoned fronts will alleviate the environmental problems at the SCB quarry, allowing to gain this deposit for a period of up to 30 years, and will facilitate the exploitation of the marl deposits below these rocks, which are currently classified as waste.

The addition of natural fluorapatite seems to be a specific asset to contribute to the solution of current and future problems related to the management of natural resources (limestone), energy saving, and environmental protection. This increases the manufacturing sustainability of SR Portland cement by reducing CO_2_ emissions from the decarbonation of CaCO_3_ (from limestone) and combustion (petroleum coke).

As perspectives, this study can be completed by:

• making an energy study of estimated SR raw meal combinations in order to optimize its energy consumption
• manufacturing optimized SR Portland cement raw meals at laboratory and industrial scales.

## Data Availability

The original contributions presented in the study are included in the article/Supplementary Material, further inquiries can be directed to the corresponding authors.

## Abbreviations

C, CaO; S, SiO_2_; A, Al_2_O_3_; F, Fe_2_O_3_; N, Na_2_O; K, K_2_O; $\bar{S}$ , SO_3_; P, P_2_O_5_; C_3_S, Ca_3_SiO_5_; C_2_S, Ca_2_SiO_4_; C_3_A, Ca_3_Al_2_O_6_; C_4_AF, Ca_4_Al_2_Fe_2_O_10_; C_2_F, Ca_2_Fe_2_O_5_; CH, Ca (OH)_2_; LSF, Lime saturation factor; SIM, Silica ratio; ALM, Alumina ratio; L.P, Liquid phase; LOI, Loss in ignition; NAF, Natural fluorapatite.
